# Research on Characterization of Nylon Composites Functional Material Filled with Al_2_O_3_ Particle

**DOI:** 10.3390/polym15102369

**Published:** 2023-05-19

**Authors:** Jibing Chen, Bowen Liu, Maohui Hu, Qianyu Shi, Junsheng Chen, Junsheng Yang, Yiping Wu

**Affiliations:** 1 School of Mechanical Engineering, Wuhan Polytechnic University, Wuhan 430023, China; kysdmbylbw@163.com (B.L.); hmhwhqgdx@163.com (M.H.); 15965826765@163.com (Q.S.); chenjs9610@163.com (J.C.); yangjunsheng2008@163.com (J.Y.); 2 School of Materials Science and Engineering, Huazhong University of Science and Technology, Wuhan 430074, China; ypwu@mail.hust.edu.cn

**Keywords:** PA6/Al_2_O_3_ composite, functional material, microstructure, mechanical property

## Abstract

This study revolves around the issues raised by the current semiconductor device metal casings (mainly composed of aluminum and its alloys), such as resource and energy consumption, complexity of the production process, and environmental pollution. To address these issues, researchers have proposed an eco-friendly and high-performance alternative material—Al_2_O_3_ particle-filled nylon composite functional material. This research conducted detailed characterization and analysis of the composite material through scanning electron microscopy (SEM) and differential scanning calorimetry (DSC). The results show that the Al_2_O_3_ particle-filled nylon composite material has a significantly superior thermal conductivity, about twice as high as that of pure nylon material. Meanwhile, the composite material has good thermal stability, maintaining its performance in high-temperature environments above 240 °C. This performance is attributed to the tight bonding interface between the Al_2_O_3_ particles and the nylon matrix, which not only improves the heat transfer efficiency but also significantly enhances the material’s mechanical properties, with a strength of up to 53 MPa. This study is of great significance, aiming to provide a high-performance composite material that can alleviate resource consumption and environmental pollution issues, with excellent polishability, thermal conductivity, and moldability, which is expected to play a positive role in reducing resource consumption and environmental pollution problems. In terms of potential applications, Al_2_O_3_/PA6 composite material can be widely used in heat dissipation components for LED semiconductor lighting and other high-temperature heat dissipation components, thereby improving product performance and service life, reducing energy consumption and environmental burden, and laying a solid foundation for the development and application of future high-performance eco-friendly materials.

## 1. Introduction

With the miniaturization, integration, high-density, and high-speed development of electronic components, heat dissipation has become a very serious problem [1], especially as various light-emitting diodes (LEDs) are widely used in various places for urban lighting. High-power and high-brightness LEDs generate a tremendous amount of heat, making it challenging to maintain the optimal operating temperature range of 25–30 °C for LEDs. Traditional inorganic thermally conductive materials such as metals and ceramics can no longer meet the needs of further development of electronic products, and new high-efficiency thermally conductive materials must be used to dissipate heat promptly [2]. Polymer-based composite materials refer to the introduction of one or more inorganic or organic materials with specific properties into the polymer matrix through various processing methods (including chemical and physical means) so that the polymer also has some special properties, such as thermal conductivity, shielding of electromagnetic waves, dielectric properties, electrical conductivity, and excellent damping performance [3]. Thermally conductive polymer composites have excellent thermal conductivity and unparalleled molding processability, electrical insulation, and corrosion resistance of traditional inorganic thermally conductive materials [4], becoming a research hotspot in the field of polymer materials.

The thermal conductivity of metals mainly relies on freely moving electrons, and the heat transfer mechanism is the formation of a heat conduction band using unconstrained electrons that can move freely; the thermal conductivity of inorganic non-metallic materials can only be conducted through the microscopic structure of mutual contact between material molecules by transferring phonons, using phonons as heat transfer carriers to transfer heat. Phonons are mechanical waves generated by lattice vibrations in crystals (mainly vibrations of atoms, molecules, and groups) [5,6], which are successively transferred between material molecules, so their thermal conductivity efficiency is not as good as that of free electrons. In contrast, polymers themselves are composed of molecular chains with very large molecular weights, and it is difficult to form a free electron energy band inside the material. Therefore, polymers mainly transfer heat through mutual contact between microscopic structures. However, unlike the phonon thermal conductivity of inorganic non-metallic materials, polymers themselves are composed of mixtures of homologous substances with different molecular weights, with larger van der Waals forces between molecular chains and longer chains that are prone to entanglement at the ends, resulting in more defects between the molecules. Therefore, polymer materials are particularly prone to phonon scattering, leading to low heat transfer efficiency and poor thermal conductivity of polymer materials [7]. Currently, there are two main approaches to preparing thermally conductive composite materials [8]: intrinsically thermally conductive polymer composites with inherent heat transfer capabilities, and filler-based thermally conductive polymer composites using externally added fillers. Intrinsic thermally conductive polymer materials have a large number of conjugated structures in their molecular chains and a relatively large proportion of crystalline regions, and these special structures give the polymer itself good heat transfer capabilities [9]. When phonons propagate in these polymers, there is less phonon scattering and hindrance, making intrinsic thermally conductive polymer composites excellent thermal conductors. However, the processing technology of intrinsic thermally conductive polymers is very complex, from the selection of initiators and the initiation of active polymer monomers, to the influence of reaction temperature and reaction time. These factors make the preparation of intrinsic thermally conductive polymers extremely difficult and the industrial production and processing difficult [10]. Therefore, based on the modern Fourier solid heat conduction theory, inorganic powders with strong heat transfer capabilities are filled into polymers to prepare high thermally conductive base polymer materials that meet the needs of the actual operating environment.

At present, a large number of studies have been conducted both domestically and abroad, aiming to develop composite functional materials with high thermal conductivity. Joao Paulo Berenguer, Arielle Berman, and other scholars [11] studied the effects of polyethylene fillers on the thermal conductivity, mechanical properties, and processability of all-polymer high thermal conductivity composite materials. Through thermal conductivity tests, tensile tests, bending tests, and impact tests, the results showed that optimizing the mass fraction and dispersion degree of polyethylene fillers can achieve a comprehensive optimization of the thermal conductivity, mechanical properties, and processability of all-polymer high thermal conductivity composite materials. Guorui Zhang, Sen Xue, and others [12] developed a high-efficiency thermal interface material with anisotropic orientation and high vertical thermal conductivity, by adjusting the distribution and orientation of graphene fillers, improving the thermal management performance of microelectronic devices, extending device life, and improving energy efficiency. You Y-L, Li D-X, and others [13] prepared GF/PA6 composite materials containing different solid lubricants in the laboratory, and used a ball-disc friction tester to evaluate the friction and wear performance of different composite materials, revealing the mechanism of various solid lubricants in reducing friction and wear. One paper [14] mainly studied the thermal conductivity of polymer composite materials based on phenolic resin and boron nitride (BN), by adding boron nitride fillers to phenolic resin, improving the thermal conductivity of composite materials, and evaluating the thermal conductivity, thermal stability, and microstructure of composite materials through thermal conductivity tests, thermogravimetric analysis, and scanning electron microscopy. Another [15] analyzed the microstructure, thermal stability, and mechanical properties of Al_2_O_3_ and SiC-reinforced PA6 hybrid composite materials, and the experimental results showed that Al_2_O_3_ and SiC particles were uniformly distributed in the PA6 matrix, effectively improving the mechanical properties of the materials. Jiaqi Zhang, Xianzhao Jia, and others [16] mainly studied the effects of additive fluoroelastomer (FVMQ) and Al_2_O_3_ particle co-filling on its mechanical properties, thermal properties, and friction properties at high temperatures, verifying that by optimizing the mass fraction and dispersion degree of Al_2_O_3_ particle fillers, a comprehensive optimization of the mechanical properties, thermal properties, and friction properties of fluoroelastomer composite materials under high-temperature conditions can be achieved. Qiu Hong Mu, Dan Peng, and other scholars [17] studied the effect of filling Al_2_O_3_ particles on the thermal conductivity of silicone rubber, and evaluated the thermal conductivity, thermal stability, and microstructure of composite materials through thermal conductivity tests, thermogravimetric analysis, and scanning electron microscopy. One work [18] explored the effects of filling Al_2_O_3_ compounds on the thermal conductivity and rheological properties of epoxy resin and liquid crystal epoxy resin composite materials, by adding Al_2_O_=_ compound fillers to epoxy resin and liquid crystal epoxy resin, improving the thermal conductivity and rheological properties of composite materials. Konopka K, Krasnowski M, and others [19] used pulsed plasma sintering (PPS) method to prepare Al_2_O_3_ samples and NiAl-Al_2_O_3_ composite materials, and analyzed the microstructure, phase composition, thermal stability, and mechanical properties of the two materials by X-ray diffraction (XRD), thermogravimetric analysis (TGA), and differential scanning calorimetry (DSC), and evaluated the significant influence of pulsed plasma sintering parameters on the microstructure and mechanical properties

According to the literature cited above, a series of research work has been carried out on thermal conductive materials for semiconductor devices. In order to ensure that these thermal conductive materials have excellent comprehensive performance, researchers usually choose to fill polymer materials with high thermal conductivity inorganic fillers or metal fillers. The thermal conductive materials prepared in this way have the advantages of low cost and easy processing. Through appropriate physical and chemical treatment methods or adjusting the experimental formula ratio, such thermal conductive materials can meet the specific application scenarios with thermal conductivity requirements. PA6 has good mechanical properties, thermal stability, and chemical stability, and can adapt to various complex working environments. More importantly, PA6 has high adjustability, and its performance can be improved by adding different types and proportions of fillers, reinforcements, and auxiliaries. Therefore, this study chose to add inorganic fillers (Al_2_O_3_) to nylon (PA6) and add 5% diethylhexyl phthalate (DEHP) in a certain proportion to prepare materials with specific functions. In order to study the mechanical properties of these functional materials, various instruments such as scanning electron microscopy (SEM) (JSM-7500F, JEOL Ltd., Tokyo, Japan), differential scanning calorimeter (DSC) (1/700 type, Mettler-Toledo, Zurich, Switzerland), and thermal conductivity meter (DTC-300, TA Instruments, New Castle, DE, USA) were used for testing and analysis. The results show that this high thermal conductivity functional material has great potential to replace metal shell materials and can be used to manufacture semiconductor devices with a metal appearance. This material performs excellently in polishing performance, thermal conductivity, and molding performance, providing promising possibilities for replacing metal materials. For example, in LED semiconductor lighting, it can be used as an effective heat dissipation material.

## 2. Materials and Methods

### 2.1. Raw Materials

The thermally conductive base material nylon (PA6) was purchased from Zhuhai SMIKA Polymer Company (Zhuhai, China); the thermally conductive filler Al_2_O_3_ (purity ≥ 99%) was obtained from National Medicine Group Chemical Reagent Co., Ltd. (Shanghai, China); the coupling agent diethylhexyl phthalate (DEHP) was provided by Foshan New Material Technology Co., Ltd. (Foshan, China).

### 2.2. Preparation of Materials

Initially, the nylon plastic granules underwent essential pretreatment. The nylon granules were dried in an oven at a temperature of 120 °C for 24 h to eliminate moisture, ensuring the stability of material performance in subsequent experiments.

In this study, we conducted three sets of experiments. Firstly, to achieve a good thermal conductivity, thermal stability, and mechanical properties, we combined the research from ref. [20,21] and prior theoretical analysis. Nylon PA6, thermal conductive filler Al_2_O_3_, and DEHP were mixed at a mass ratio of 80:18:2. A high-speed stirrer was used to ensure that all components were fully mixed and formed a homogeneous mixture. Subsequently, the obtained mixture was extruded using an extruder to give the material a certain shape. The extruded material was then dried at 80 °C for 2 h to remove any residual moisture, ensuring the smooth progress of the subsequent injection molding process.

Secondly, with the aforementioned experimental procedure kept consistent, we conducted grouped experiments with filler particle size, filler shape, and filler ratio as variables to evaluate the thermal performance of the material. The particle sizes selected were 5 μm, 20 μm, and 100 μm; the filler shapes were spherical and flaky; the filler ratios were 10%, 20%, 30%, 40%, and 50%.

Lastly, to analyze the relationship between the filler ratio and mechanical properties in more detail, we set up nine groups of gradient experiments for the filler ratio, namely 0%, 5%, 10%, 15%, 20%, 25%, 30%, 35%, and 40%, while maintaining the aforementioned experimental procedure.

### 2.3. Characterization of Thermal Conductivity

After the experiment was completed, we immersed the samples used for testing thermal performance in liquid nitrogen, allowing them to rapidly solidify and become brittle at low temperatures, thus facilitating subsequent cross-sectional observation and analysis. Then, the cross-section of the sample was placed on conductive adhesive and a sputter coating treatment performed to provide good conductivity and adhesion for observation and analysis under a scanning electron microscope (SEM). The SEM was used to observe the distribution characteristics and morphology of the thermally conductive filler in the matrix material. Finally, the prepared heat-resistant plastic samples were sent to a differential scanning calorimeter (DSC) for heat resistance performance analysis. We sought to understand the thermal stability and thermal degradation behavior of the composite material at different temperatures, thereby evaluating its application potential in high-temperature environments. Furthermore, we employed a thermal conductivity meter to analyze the samples. By applying heat to the sample and measuring its temperature change over a certain period, we calculated the sample’s thermal conductivity, which allowed us to predict the material’s thermal management performance in practical applications.

### 2.4. Mechanical Analysis

In this study, tensile tests were conducted to evaluate the mechanical properties of the composite material, using an electronic universal testing machine (WDT-10, Shenzhen Kaiqiangli Experimental Instrument Co., Ltd., Shenzhen, China.) with a testing temperature of 25 °C. To ensure the accuracy and comparability of the experiments, the preparation and testing process of the tensile samples followed the relevant provisions of ASTM D638. Meanwhile, the size of the samples referred to the ISO 527-2 Type 1A standard requirements to ensure that the shape and dimensions of the samples met internationally accepted requirements. In the actual testing process, the samples were stretched at a transverse speed of 25 mm/min to simulate the loading speed and conditions that may be encountered in real-life applications. Finally, the American TA Instruments (New Castle, DE, USA) Q800 analyzer was used for single cantilever mode testing, gaining a deeper understanding of the mechanical properties of the composite material and its potential in various application scenarios.

### 2.5. Scanning Electron Microscopy (SEM)

A scanning electron microscope (SEM) was used to study the microstructure of PA6, Al_2_O_3_, and the composite material. Observing the microstructure and interactions within PA6, Al_2_O_3_, and their composite material helps to reveal the microstructural characteristics of the samples.

## 3. Results and Discussion

### 3.1. Microstructure Analysis of Thermal Polymer Materials

**Figure 1:**
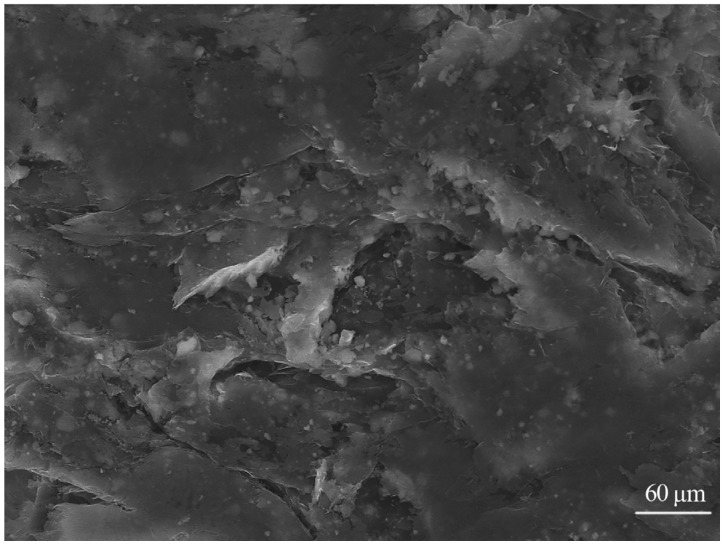
PA6 material microstructure image.

**Figure 2:**
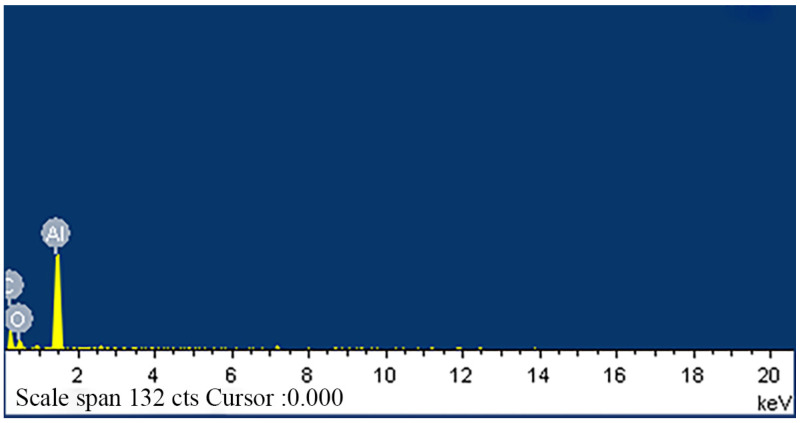
Al_2_O_3_ energy spectrum analysis image.

In order to measure the thermal conductivity, the test samples were first treated with liquid nitrogen to increase the brittleness of the material. Subsequently, the cross-section of the samples was placed on a conductive adhesive and subjected to sputter coating, enabling observation of the distribution characteristics and morphology of the thermally conductive fillers within the matrix material under a scanning electron microscope (SEM). Following this step, we utilized a differential scanning calorimeter (DSC) to analyze the thermal stability of the heat-resistant plastic samples to understand their performance under high-temperature conditions. Prior to the formal preparation of thermally conductive polymer materials, a detailed examination of the microstructure and energy spectrum distribution of the fillers and PA6 was conducted using a scanning electron microscope, based on the composition ratio of the thermally conductive fillers. The results are shown in Figure 1 and Figure 2.

**Figure 3:**
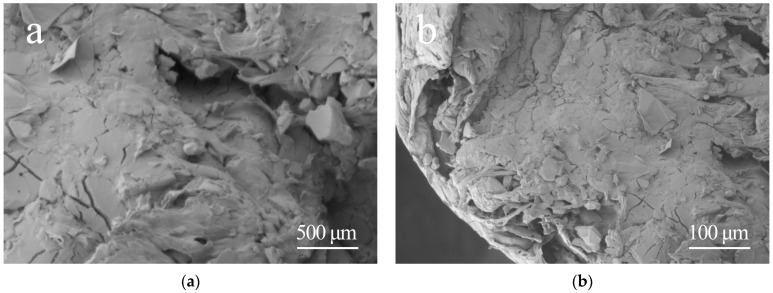
**Figure 3.** SEM photographs of nylon composites: (**a**) 500 μm; (**b**) 100 μm.

After successfully preparing the thermally conductive composite material, its microstructure was observed using a scanning electron microscope (SEM), as shown in Figure 3. Comparing Figure 1 and Figure 3, it can be found that the SEM image of the original PA6 displays a relatively uniform and smooth surface, while the SEM image of the PA6 composite material with added Al_2_O_3_ filler exhibits a clear distribution of filler particles and an interfacial region is formed between the Al_2_O_3_ filler and the PA6 matrix. Furthermore, the thermally conductive filler (Al_2_O_3_) appears as flake-like or spherical shapes and is relatively uniformly distributed within the nylon matrix material, which is beneficial for forming continuous thermal conduction paths or networks within the composite material. After the addition of the thermally conductive filler, the original microstructure of the matrix material changes, resulting in the thermally conductive composite material exhibiting excellent thermal conductivity performance.

### 3.2. DSC Testing

**Figure 4:**
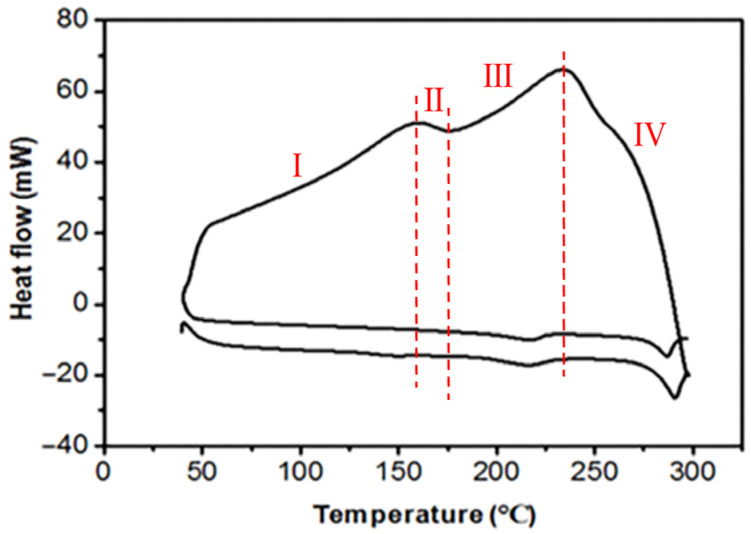
DSC curves of thermally conductive polymers. (Ⅰ) Glass transition phase; (Ⅱ) Thermal stabilization phase; (Ⅲ) Melting phase; (Ⅳ) Crystallization phase.

In this study, the thermal properties of the thermally conductive polymer samples were analyzed using a differential scanning calorimeter (DSC). During the experiment, the samples were placed in a nitrogen atmosphere and heated at a rate of 20 °C per minute, with the temperature increasing from room temperature to 300 °C. After reaching 300 °C, the temperature was maintained at 10 °C for 10 min, followed by cooling. The obtained DSC curve of the samples is shown in Figure 4. Based on the results in Figure 4, the entire DSC curve can be divided into four stages [22]. In the first stage, from the start of heating to approximately 160 °C, the DSC curve gradually rises and reaches the peak of the first endothermic peak; at this temperature, the polymer transitions from a glassy state to a highly elastic rubber-like or viscous fluid state, and the motion of the molecular chains becomes more flexible. The subsequent second stage extends from the end of the 160 °C endothermic peak to a temperature of 175 °C. The third stage has a temperature range of 175 °C to 240 °C, where the curve starts to rise, ultimately forming the second endothermic peak; at this temperature, the crystalline regions of PA6 begin to melt, and the movement of the molecular chains further increases. The final fourth stage covers a temperature range of 230 °C to 300 °C, during which the DSC curve begins to decline and exhibits a faster descent rate; in this process, heat is released, and the polymer material’s chain segments begin to rearrange and form crystalline regions.

### 3.3. Thermal Conductivity Testing

**Figure 5:**
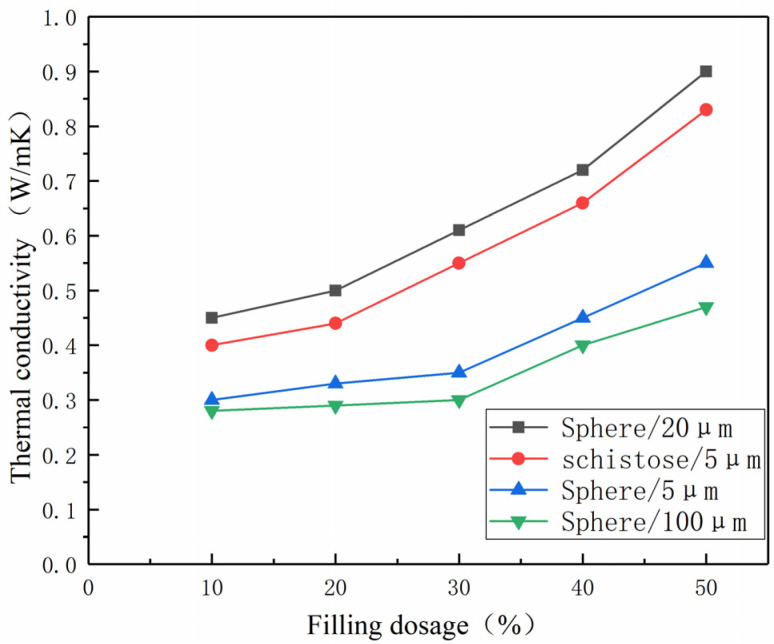
The thermal conductivity of spherical and flaky Al_2_O_3_ filled with nylon composites with 5, 20, and 100 μm particles.

In this project, we conducted experiments on the thermal conductivity of polymer materials prepared based on Al_2_O_3_ with different diameters and morphologies. The diameters of the fillers used were 5 μm, 20 μm, and 100 μm, and the morphologies included spherical and flake-like shapes. Figure 5 shows the corresponding experimental data curves. Combining the experimental data with the literature [23], several conclusions can be drawn: (1) Under different diameters or different morphologies of the filler, the thermal conductivity of the polymer materials exhibits an increasing trend, indicating that the thermal conductivity of nylon composites increases with the increase in the content of Al_2_O_3_ filler. This phenomenon can be attributed to the increase in filler content, which helps form thermal conduction paths and networks in the composite material, thereby improving thermal conductivity; (2) By observing the data curves of the three groups with filler diameters of 5 μm, 20 μm, and 100 μm, it can be concluded that the thermal conductivity is the highest when the filler diameter is 20 μm and the lowest when the filler diameter is 100 μm. This indicates that within a specific particle size range, the thermal conductivity of the composite material increases with the increase in particle size; however, beyond this range, the thermal conductivity gradually decreases. This phenomenon may be due to the larger particle size filler reducing the thermal conductivity of the composite material, leading to a decrease in overall thermal conductivity; (3) Comparing the data of spherical and flake-like fillers with a diameter of 5 μm, it can be found that the flake-like fillers are more effective in improving the thermal conductivity of the composite material than spherical fillers.

### 3.4. Mechanical Properties Testing

**Figure 6:**
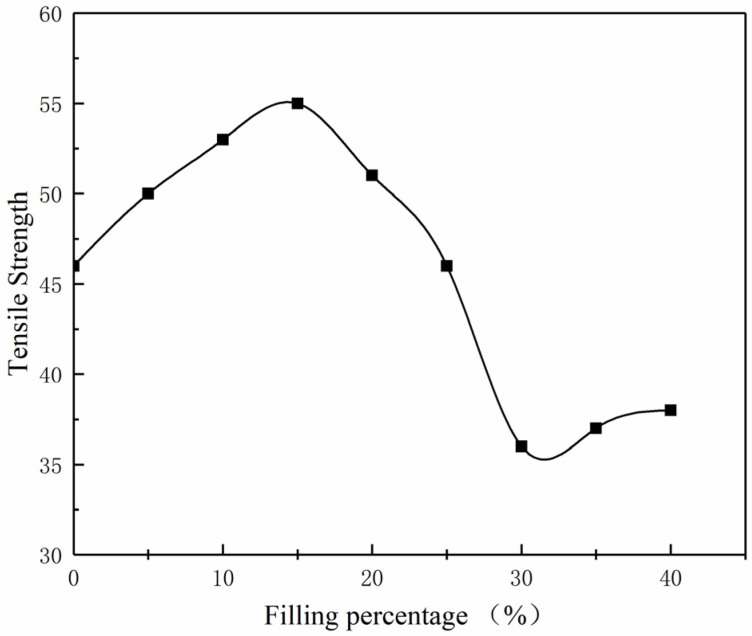
Tensile strength curves of alumina/nylon composites. ■ denotes the tensile strength at various filler percentages.

Figure 6 shows the change curve of the tensile strength of composite materials as the content of Al_2_O_3_ filler increases. According to the experimental data curve, the tensile strength of nylon is 46 MPa. When the alumina content reaches 14%, the tensile strength of the composite material reaches its maximum value of 55.1 MPa, which is about 19% higher than that of pure nylon material. However, when the alumina filler content exceeds 14%, the tensile strength of the composite material significantly decreases, showing an inverse relationship with the proportion of filler. When the alumina content reaches 30%, the tensile strength is 35.5 MPa. This observation indicates that the tensile strength of the composite material increases with the increase in alumina content within a specific range (alumina content below 10%). When the content is below this range, its tensile strength is even lower than that of single nylon material.

**Figure 7:**
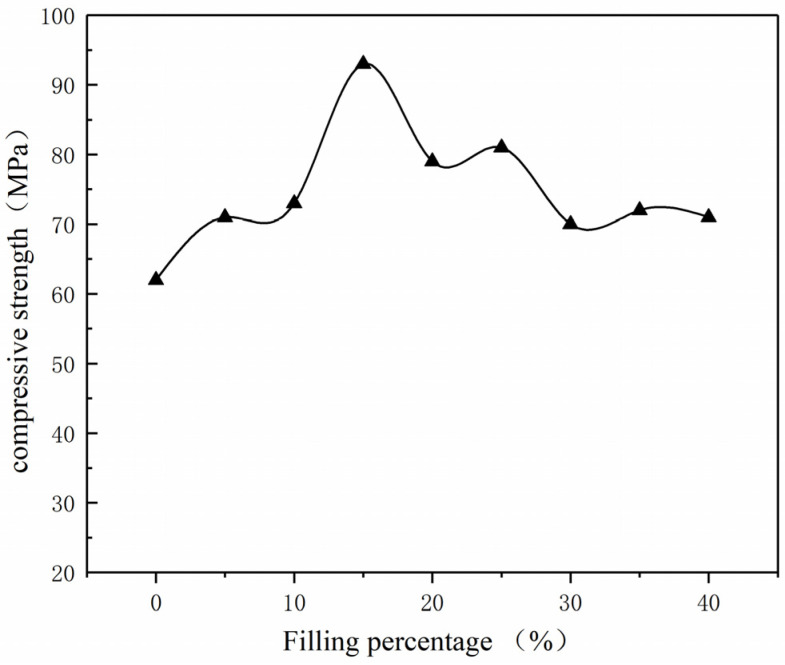
Compressive strength curve of Al_2_O_3_/nylon composites. ▲ denotes the compressive strength at various filler percentages.

With the addition of alumina filler, the compressive strength of nylon composite materials has been improved accordingly. An electronic universal testing machine was used to measure the compressive strength of composite materials, and the experimental results are shown in Figure 7. When the alumina content reaches 15%, the compressive strength of the composite material reaches its highest value of 94 MPa. Compared with pure PA6, the compressive strength of this composite material has increased by 47.6%. Within a certain range (alumina content ≤ 15%), the compressive strength of nylon composite materials increases with the increase in alumina content. This trend can be attributed to the dispersion of alumina particles in the polymer matrix and the interaction between alumina particles and the polymer matrix [24]. However, when the alumina content exceeds this threshold, the excessive filler content leads to particle aggregation in the polymer matrix, which affects the overall performance of the composite material, resulting in a decreasing trend in compressive strength.

## 4. Conclusions

In this paper, researchers successfully prepared a composite material with high thermal conductivity using high thermal conductive functional materials, namely polyamide 6 (PA6) and alumina (Al_2_O_3_). Detailed experimental studies were conducted on the thermal conductivity, heat resistance, and performance at different filler ratios of this composite material. The experimental results show that: (1) using spherical fillers with a diameter of 20 μm can achieve a larger thermal conductivity. When the filler size is reduced to 5 μm, the thermal conductivity reaches the highest value of 0.9 W·(mK)^−1^. In addition, this composite material has good heat resistance performance and can maintain stability at the highest temperature of 240 °C. (2) The composite material has significant mechanical properties. When the Al_2_O_3_ content is 10%, the tensile strength of the thermal conductive functional material reaches its highest value. However, when the additive amount is increased to 14%, the compressive strength of the composite material reaches the highest value of 55.1 MPa, indicating that to a certain extent, increasing the Al_2_O_3_ content can improve the mechanical properties of the composite material. In summary, this high thermal conductive functional material prepared from PA6 and Al_2_O_3_ not only has excellent thermal conductivity and heat resistance performance but also has good mechanical properties. This gives this composite material a wide range of application potential in high-temperature application fields, such as aerospace, automotive manufacturing, and electronic devices. This research provides beneficial practical experience for engineers and scientists in related fields, helping to develop higher performance composite materials in actual applications.

## Data Availability

Not applicable.
